# Review of "A realistic strategy for fighting malaria in Africa" by William Jobin

**DOI:** 10.1186/1756-3305-3-68

**Published:** 2010-08-09

**Authors:** Bart GJ Knols

**Affiliations:** 1 Division of Infectious Diseases, Tropical Medicine & AIDS, Academic Medical Center, F4-217, Meibergdreef 9, 1105 AZ Amsterdam, The Netherlands

## Review

The latest World Malaria Report (2009) brought much good news in terms of the global progress in malaria control. So did recent reviews on the changing burden of malaria in Africa and the progress achieved since the signing of the Abuja declaration in 2000. Optimism reigns to such extent that the words 'elimination' and 'eradication' are now liberally used within the global malaria community.

Jobin's book, however, serves as a contrasting wake-up call, and tries to shed light on what he claims to be '*the mess we are currently making in Africa*'. With a wealth of experience, built up over 45 years of involvement in operational malaria control campaigns, Jobin provides a frank and thought-provoking opinion of the current attempts to control the disease, and is (negatively) outspoken about the roles therein of major players like the PMI, WHO, and USAID, all of whom Jobin worked for during some stage of his career. Accordingly, Jobin speaks of the need '*to inject realism into current naive and ephemeral efforts to control malaria in Africa*'. He argues strongly against the renewed eradication euphoria, calls vaccine developers 'dreamers', and tells us that malaria is here to stay. The best we can do is to be clever and reduce it to very low levels over prolonged periods of time. So what does *his *realistic strategy look like?

First, Jobin urges us to learn from past experiences. That it took six decades to eliminate malaria from Italy, that malaria control *sec *is not sufficient to remove it permanently, and that historic attempts to eliminate malaria in Africa (notably the Garki project in Nigeria and the Gezira project in Sudan) ended in dismal failure. The maths clearly showed that, even with massive reductions in mosquito biting density, transmission cannot be permanently disrupted. For Sudan, it was cessation of funding that resulted in a massive epidemic after a decade in which malaria was virtually eliminated. The classic mistakes made by PMI before, during, and after the IRS programmes that were staged in Angola and subsequently Zanzibar are telling examples that, as recent as five years back, there was no learning from past mistakes. Malaria control can be summarised as: Aid money arrives, intense (spraying, nets) campaigns bring prevalence down to <1%, aid dries up, malaria comes back. Malaria control in Africa is like a trampoline.

Secondly, Jobin uses example after example of badly designed malaria control programmes, with minimal baseline data, lack of evidence-driven action plans, and insufficient quality control. Here he lashes out at WHO, RBM and the GF. Only with a thorough and basic approach to impact evaluation, he argues, will control campaigns come to bear fruit over prolonged periods of time.

Thirdly, there is a strong need to revive training centres for malaria personnel if endemic countries are to set their own tailor-made agenda to control malaria. Such capacity building should shield NMCPs from the agenda-driven influences by multiple small or large NGOs that flood endemic nations with their preferred mode of attack.

Jobin argues that more permanent solutions are within reach but not on the radar of donor organisations. Simple improvements to houses (screens and improved air circulation) can make a house less prone to mosquito attack whilst at the same time increasing comfort for its occupants. Larval source management, although gaining more interest, is still only used on a marginal scale but should complement adulticidal methods. Integration of tools, or the 'Goat cheese strategy' as Jobin names it, is likely to yield much more benefit than single interventions. Disbursement of gradually increasing amounts of money to sustain a realistic control programme, based on evidence, may be much better than throwing millions up front into a programme that may lead to dismal failure after the purse is empty. Staying away from dictatorship regimes and corrupt nations concludes the list.

This is all pretty harsh news for the optimists of today, but the statement that came out of a workshop organised at Yale University in 2008 deserves mention here (and mentioned in Jobin's book): 'There should be no illusion of rapid success against malaria because unrealistic targets and unsustainable goals carry the dangers of fatalism and the abandonment of effort. Once begun, the campaign must be sustained because otherwise there is the risk that, by compromising acquired immunity, temporary but unsustainable advances could transform chronic malaria into devastating epidemics. Strategies should be developed into long-term public health efforts rather than dramatic but temporary interventions'.

Jobin ends his book with a plea to develop specific tailor-made malaria control programmes for the (5) different ecological zones in Africa and with setting up training centres (for English, French, and Portuguese speaking countries) to develop the capacity to run campaigns and monitor and evaluate the impact thereof. He sums it all up in a final sentence: 'So let us not be carried away by illusions and fantasies. Let us begin in one country at a time, realistically, carefully, and deliberately, to reduce malaria in Africa. And let us begin now, in South Africa, Senegal, Mozambique, and Tanzania'.

Whether or not Jobin's book will become the wake-up call for PMI, WHO, and the GF remains to be seen. As seen so often, lonely voices that go against commonly accepted practice and policies crafted by bureaucrats in large organisations tend to be ignored. It is hoped though that, even if they do not act according to the realistic strategy proposed here, that at a minimum they would take the time to soak up the compelling arguments throughout this book. There are certainly a few lessons to be learned.

## Abbreviations

GF: Global Fund to fight AIDS, tuberculosis, and malaria; IRS: Indoor Residual Spraying; NGO: Non-governmental organisation; NMCP: National Malaria Control Programme; PMI: President's Malaria Initiative; RBM: Roll Back Malaria; WHO: World Health Organization.

## Competing interests

The author declares that he has no competing interests. He is a member of the Editorial Board of *Parasites & Vectors*.

